# Understanding the landscape for driving antimicrobial stewardship implementation at scale in South Africa

**DOI:** 10.1093/jacamr/dlag232

**Published:** 2026-10-22

**Authors:** Dena Van Den Bergh, Esmita Charani, Candice Bonaconsa, Adrian Brink, Nicolette Du Plessis, Angeliki Messina, Malcolm Miller, Natalie Schellack, Hafsah Tootla, Klaus B Von Pressentin, Marc Mendelson

**Affiliations:** Division of Infectious Diseases and HIV Medicine, Department of Medicine, Groote Schuur Hospital, University of Cape Town, Cape Town, South Africa; Division of Infectious Diseases and HIV Medicine, Department of Medicine, Groote Schuur Hospital, University of Cape Town, Cape Town, South Africa; Division of Infectious Diseases and HIV Medicine, Department of Medicine, Groote Schuur Hospital, University of Cape Town, Cape Town, South Africa; Division of Medical Microbiology, Faculty of Health Sciences, University of Cape Town, Cape Town, South Africa; National Health Laboratory Service, Groote Schuur Hospital, Cape Town, South Africa; Institute of Infectious Disease and Molecular Medicine, Faculty of Health Sciences, University of Cape Town, Cape Town, South Africa; Department of Paediatrics, University of Pretoria, Pretoria, South Africa; Digital Medication Management, Netcare Group Ltd, Johannesburg, South Africa; Department of Anaesthesia and Perioperative Medicine, University of Cape Town, Cape Town, South Africa; Department of Pharmacology, University of Pretoria, Pretoria, South Africa; Division of Medical Microbiology, Faculty of Health Sciences, University of Cape Town, Cape Town, South Africa; National Health Laboratory Service, Groote Schuur Hospital, Cape Town, South Africa; Division of Family Medicine, Department of Family, Community and Emergency Care, University of Cape Town, Cape Town, South Africa; Division of Infectious Diseases and HIV Medicine, Department of Medicine, Groote Schuur Hospital, University of Cape Town, Cape Town, South Africa

## Abstract

**Background:**

Antimicrobial stewardship (AMS) programmes are essential for responding to antimicrobial resistance, yet implementation at scale remains challenging everywhere, including in South Africa. This study explored the landscape for driving AMS implementation, identified barriers and enablers and identified priorities for subsequent multi-facility interventions.

**Methods:**

Phase 1 of a prospective, multi-phased mixed-methods study used participatory action research informed by the Breakthrough Series methodology and Theory U. Health professionals from public and private sectors participated in workshops in Cape Town and Johannesburg. Activities included mapping individual AMS journeys, identifying barriers and enablers at individual, institutional/facility and national/provincial levels, conducting a SWOT analysis and prioritizing potential AMS implementation projects. Data were analysed using descriptive content analysis.

**Results:**

Participants identified substantial enabling factors alongside persistent barriers. Key enablers included mentorship, multidisciplinary collaboration, management support, functional AMS and infection prevention structures, guidelines, diagnostics, national policies and professional society advocacy. Barriers included professional isolation, weak interprofessional relationships, limited funding and staffing, inadequate training, weak governance, poor data use, fragmented public-private and inter-provincial systems, and inconsistent policy implementation. Strengths were identified as being existing programmes and collaboration, weaknesses as resource and education gaps, opportunities as cross-sector collaboration and technology and threats as limited political support and economic constraints. Eight priority AMS areas were identified as collaborative projects for implementation across multiple healthcare settings.

**Conclusion:**

South Africa has important existing assets for AMS implementation, although these are unevenly distributed and inconsistently used. Strengthening leadership and collaboration, leveraging existing structures and implementing context-specific countermeasures may support at-scale AMS implementation.

## Introduction

After decades of increasing evidence of the spread of bacterial antimicrobial resistance (AMR) and sombre projections of its serious and devastating impact on health outcomes and health systems, implementing antimicrobial stewardship (AMS) programmes remains challenging globally, including in South Africa.^1,2,3^

A 2023 report by the WHO illustrated the scale of this challenge and showed that of the 166 countries that responded to the AMR self-assessment survey 28% (47 of 166) reported that they were implementing and monitoring their National Action Plans (NAPs) for AMR.^4^ This gap between having policy frameworks and operationalizing change is mirrored in South Africa, where AMS implementation has largely been driven not by national level structures but by individual health professionals serving as AMS champions within their facilities.^5,6,7^ Whilst this model has served as a critical contribution to AMS development and implementation, especially within hospital settings, it has limited potential for implementing AMS at the scale and pace required to make the impact needed.^6,7^ In addition, as the call to action to mitigate AMR continues to escalate, so too has the narrative on barriers to AMS implementation, particularly in low- and middle-income countries (LMICs).^8,9,10^ These narratives, while bringing much-needed attention to the challenges and resources required, influence health professionals’ willingness to champion widespread AMS implementation. They also affect efforts to test solutions within the capability and resource constraints of existing systems.

The South African Antibiotic Stewardship Programme (SAASP) is a multi-disciplinary group of infectious disease specialists, pharmacists, nurses, clinical microbiologists and infection prevention and control practitioners from public and private health sectors. It was formed in 2012 to support the implementation of AMS in South Africa and to advocate for government policy action.^11^ SAASP has provided a platform for training and development in AMS. In 2017 and 2021, under the leadership of a programme manager with experience in large-scale AMS implementation, SAASP also implemented two multi-hospital public-private sector collaborative AMS interventions using the Breakthrough Series (BTS) method.^12,13^ These studies resulted in a positive impact on appropriate antibiotic use across multiple public and private hospitals, supported a more rapid spread of evidence-based AMS interventions and generated context-specific learning for AMS implementation.^12,13,14^ A 2026 systematic review on stewardship and implementation internationally found that almost 70% of studies used single site research designs and called for more rigorous multi-site research to better understand how AMS can be implemented and sustained at scale.^15^ Evidence from single sites reflects a bigger gap in that much of what is known about AMS implementation comes from isolated efforts often in academic centres rather than coordinated multi-site initiatives.

We recognized that strengthening and expanding AMS leadership capability for large-scale AMS implementation is an essential missing step to facilitate the spread and sustainability of AMS programmes in South Africa. Without a critical mass of leaders skilled to drive and scale AMS across the public and private sectors, efforts to manage AMR will remain limited and unsustainable.

Calls for strengthening AMS implementation, especially in low and middle-income countries (LMIC) emphasize the importance of strengthening leadership, understanding of the contextual factors that shape AMS implementation and adopting strategies grounded in local realities.^5,16,17^ In response to this, SAASP initiated a prospective mixed-methods study to expand and strengthen leadership capacity needed to drive the implementation and adoption of AMS and to generate and test solutions within existing health systems using the Breakthrough Series (BTS) and action research.^18^

## Methods

### Overall study design

We designed a multi-phased mixed methods study using the BTS collaborative methodology^18^ (Figure 1) comprising four phases: an initial phase (P1) to understand the existing landscape for leading AMS implementation, build collective will and identify project priorities; a training and design phase (P2) on the BTS methodology and change leadership; an implementation phase (P3) in which participants co-lead AMS projects across multiple facilities; and a reflective phase (P4) to capture leadership learnings and develop a framework for leading AMS implementation at scale. This report describes the findings from Phase 1 (P1).

**Figure 1.**
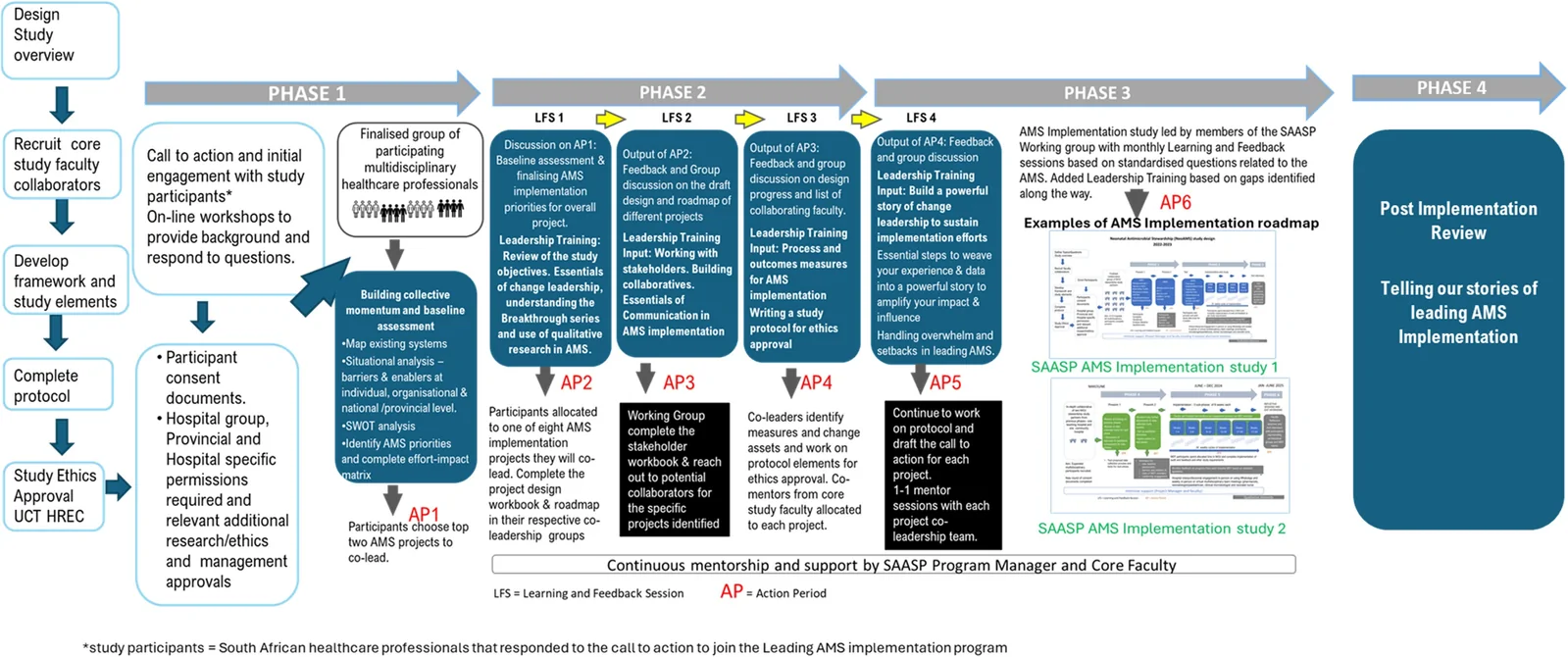
The South African Antibiotic Stewardship Programme (SAASP) collaborative breakthrough series Leading AMS implementation study.

### Recruitment

During World Antibiotic Awareness Week in November 2024, SAASP issued a call-to-action via social media and its national public-private network email list, inviting health professionals working in AMS to join a collaborative action research project. Respondents attended one of four online information workshops and were then provided with the research protocol, informed consent documents and details on the required commitment before enrolling.

### Workshop design and framework

Enrolled participants attended one of two full-day in-person participatory action research workshops in Cape Town or Johannesburg, together with ten study faculty of key AMS leaders and researchers. The workshops were co-designed with core faculty members and facilitated by the SAASP Programme Manager, an advanced facilitator trained in participatory action research and the BTS methodology with extensive experience in large-scale AMS implementation.

The workshops employed a framework based on Theory U, a collaborative approach to leading change through reflection and co-creation,^19^ incorporating 3D modelling, a graphic representation of systems and processes.^20^ The three-dimensional mapping approach supports deeper engagement, fosters shared understanding and facilitates identification of leverage points within complex healthcare systems.^20^ This enabled participants to visually map system structures and interconnections and explore alternative pathways for change.

#### Mapping AMS journeys

Participants independently mapped a timeline of their professional journey in AMS on an A3 artboard, then depicted the following six elements prompted by the facilitator on their timeline using clay or art materials: obstacles faced; skills and knowledge gained; people who supported or hindered their journey; impactful key incidents or events; energizing factors; and sources of frustration or demotivation. Participants then shared their 3D maps in small groups (four to five participants), with key highlights fed back to the larger group.

#### Identifying enablers and barriers

The participants remained in groups of four to five and, drawing on their own and colleagues’ journeys, identified and recorded enablers and barriers to AMS at three levels—individual, institutional/facility and national/provincial—using the provided visual flipchart templates. Findings from each group were shared with all participants.

#### SWOT analysis

Groups were next introduced to the SWOT (Strengths, Weaknesses, Opportunities and Threats) analysis framework^21^ and, reflecting on the enabler and barrier charts, constructed a SWOT analysis for AMS implementation in South Africa, which was again shared with the wider group.

#### Project prioritization and selection

As a final exercise, participants brainstormed AMS implementation projects relevant to the South African setting and mapped each project idea onto an effort-complexity-versus-impact matrix. Participants reviewed all the project ideas as a group and then selected their top two preferences for projects they would want to co-lead in subsequent phases of the study.

### Data collection and analysis

Data from group visual templates across both workshops were collated into Excel spreadsheets, including barrier and enabler charts at the three levels, SWOT analyses, priority project lists and co-leadership preferences. Effort-complexity versus impact matrices were consolidated into a single overall matrix. Data were analysed by the SAASP Programme Manager, with faculty support, using descriptive content analysis. Barriers, enablers and SWOT data were grouped into themes and subthemes, with supporting phrases drawn from the original data. Project matrix data and co-leadership preferences were reviewed to compile a prioritized list of projects for subsequent phases of the study.

### Ethical considerations

Ethical approval was obtained from the University of Cape Town Human Research and Ethics Committee (UCT HREC): HREC Ref.462/2026. All data collected were anonymized. Participation was voluntary and confidential, and no remuneration was offered to participants. Participating health professionals (SAASP Leading AMS study group) signed informed consent.

The Consolidated criteria for Reporting Qualitative Research (COREQ) checklist is included in Table S1 (available as Supplementary data at *JAC-AMR* Online)

## Results

### Demographics

Forty health professionals responded to the call to action, of whom 38 enrolled in the study, including 20 from the public sector and 18 from the private sector. Participants comprised 18 pharmacists, nine doctors and/or infectious diseases (ID) specialists, four clinical microbiologists, six nurses and infection prevention and control (IPC) practitioners and one dentist. The age range of participants included 16 (42.1%) between 25–34 years (early career), 16 (42.1%) between 35–49 years (mid-career) and 6 (15.8%) aged 50 years and above (late career). On enrolment, participants reported a mean of 7.9 years of active AMS experience (range 0–23 years), with only 3 (7.9%) having previously led multifacility AMS interventions. In addition to the 38 enrolled participants, 10 core study faculty members attended one of the two workshops, comprising 3 ID specialists or intensivists, 3 pharmacists, 2 clinical microbiologists, one family medicine specialist and one nurse, with a mean of 16.5 years of AMS experience (range 6–25 years), of whom 6 (60.0%) had previously led large-scale multifacility AMS initiatives.

### Enablers and barriers at different levels of the healthcare system

The thematic analysis of barriers and enablers to AMS implementation, derived from the lived experience of 48 health professionals, is shown in Table 1. At the individual level, enablers included expert mentorship, supportive multidisciplinary colleagues, a positive mindset and management support. Barriers included professional isolation, weak interprofessional relationships, colleagues’ negative attitudes and inadequate funding and staffing. At the institutional/facility level, enablers included functional AMS, IPC and pharmacy and therapeutics committees; access to guidelines and diagnostics; standardized prescription tools; and quality improvement methods. Barriers included limited resources, weak governance structures, poor data utilization and insufficient AMS and IPC training. At the national/provincial level, enablers comprised the Essential Medicines List, the National Action Plan for AMR, provincial AMS and IPC structures and professional society advocacy. Barriers included inadequate funding, public-private and inter-provincial fragmentation and weaknesses in policy implementation and currency.

**Table 1.** Thematic analysis of barriers and enablers to leading AMS implementation in South Africa derived from the lived experience of participating health professionals’ reflections of their AMS journey (n = 48)

LEVEL	ENABLERS	BARRIERS
**INDIVIDUAL** (Health professionals)	**Expert support** Access to mentors, role models and experts	**Isolation** Working alone, lone voice
	**Health professionals we work with** Inspiring colleagues, fellow peers, like-minded AMS champions, having multidisciplinary team members	**Weak interprofessional relationships** Silos, lack of trust, hierarchy, fragmented roles, poor communication channels
	**Positive mindset** Motivation, commitment, discipline, resilience, enthusiasm, collaboration, learning mentality	**Negative mindset** Fear, self-doubt, overwhelm, resistance to change, not assertive
	**Management support** Hospital management and departmental support	**Structural barriers** Limited or no funding, insufficient staff, limited time, absent or weak AMS, IPC structures
		
**INSTITUTIONAL/FACILITY** Hospital and Healthcare facilities	**Organizational structures and tools** AMS, IPC, PTC committees, doctor committees Local policies and guidelines, Access to diagnostics. Standardized antibiotic prescription charts	**Limited resources** Limited staff and funding, competing priorities, No or weak committees, Competing priorities
	**Data and technology** Access to antibiotic usage data and laboratory data IPC surveillance	**Lack of or poor use of data**
	Adoption of **Quality Improvement methods**	**Lack of training and education** in AMS and IPC
		
**NATIONAL/PROVINCIAL** Government and formal professional	**National structures, policies and tools** Essential medicines list (EML) National public sector database, National Action Plan (NAP) -although needs to be updated. public-private collaboration e.g. COVID	**Limited resources** Limited or no national or provincial funding, Competing priorities
	**Provincial structures and policies** Provincial AMS, IPC policies and committees	**Fragmentation** Provincial silos, Public-Private sector divide,
	**National professional societies championing AMS-IPC** SAASP, SASOCP, FIDSSA	**Policy weakness** Policies, guidelines and NAP need to be updated, Lack of implementation of policies

### SWOT analysis

Figure 2 presents the consolidated SWOT analysis of AMS implementation in South Africa, derived from a participatory, reflective analysis of barriers and enablers across different levels of the system.

**Figure 2.**
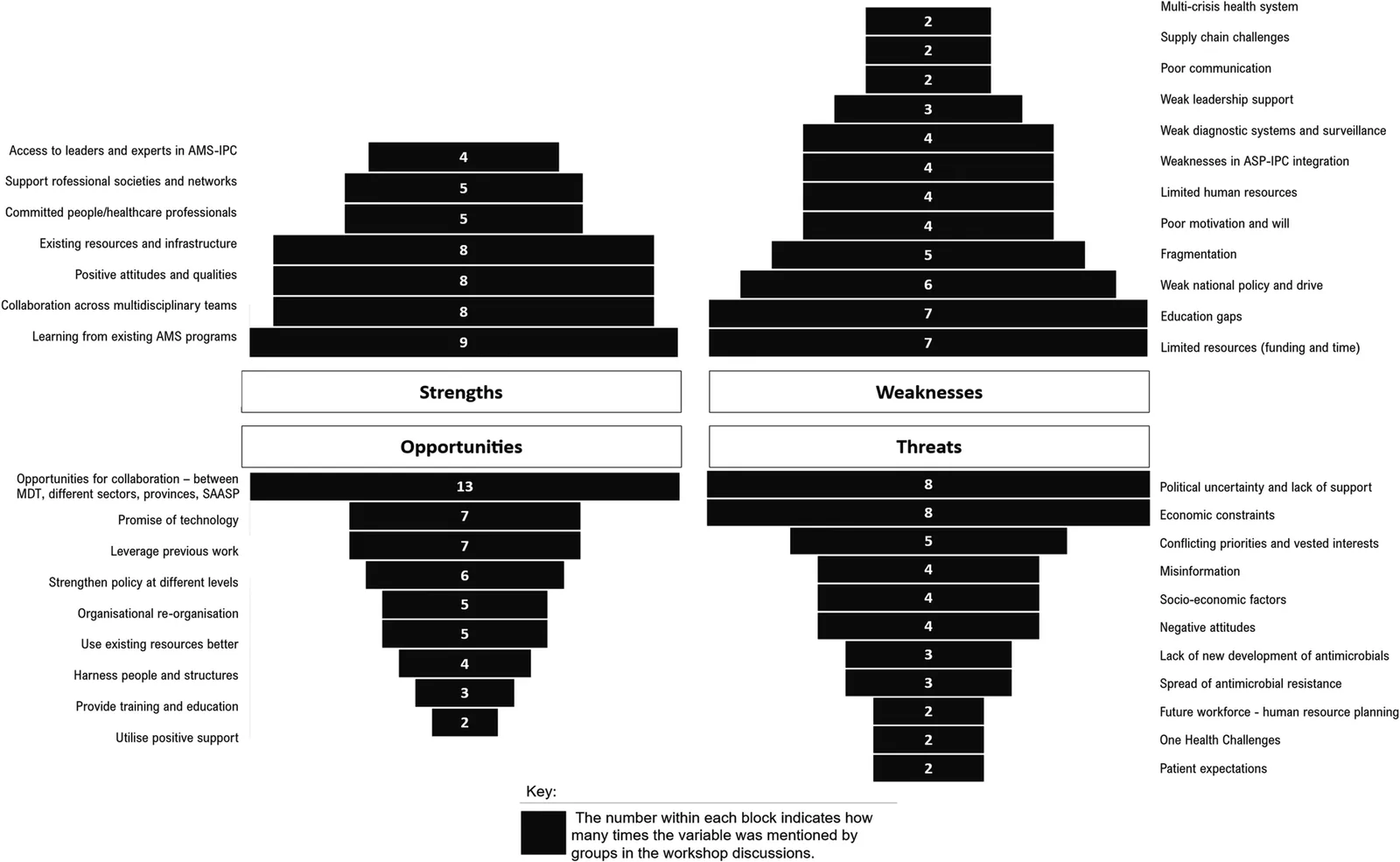
Thematic analysis of Strengths, Weaknesses, Opportunities and Threats (SWOT) to leading AMS implementation in South Africa derived through participative reflections of the barriers and enablers at individual, institutional/facility and national/provincial levels.

The top strength was being able to learn from existing AMS programmes in South Africa with multidisciplinary collaboration, positive attitudes and qualities, and existing resources and infrastructure as the next highest strengths identified within the seven themes. Of the twelve themes identified as weaknesses, limited resources (funding and time) and education gaps were the top weaknesses, followed by weak national policy and fragmentation. The opportunity for collaboration between multidisciplinary teams, different sectors, provinces and within SAASP was seen as the most important opportunity. In addition, the promise of technology, leveraging previous work done in South Africa, strengthening policy at different levels (local, provincial and national), and the opportunity to use existing resources better were amongst the other most common opportunities of nine identified. The key threats included a lack of political support and economic constraints followed by conflicting priorities, misinformation, socio-economic factors and negative attitudes.

The participants identified 14 AMS implementation projects. The analysis of the top two choices of each participant highlighted eight priorities in the following AMS categories: surgical prophylaxis, antifungal, paediatric, dental and diagnostic stewardship, carbapenem-appropriate use, nursing AMS bundle and primary care AMS (Table 2).

**Table 2.** Prioritized AMS Projects SAASP Leading AMS implementation study

	Surgical Prophylaxis	Antifungal Stewardship	Paediatric Stewardship	Diagnostic Stewardship	Carbapenem Appropriate Use	Nursing AMS Bundle	Primary Care AMS	Dental Stewardship
**Project Topic**	Improving procedure-specific adherence to guidelines and IPC bundle (C-section, joint replacement)	Pharmacist-led antifungal stewardship bundle for echinocandins	Co-designing a paediatric AMS-IPC bundle for lower respiratory tract infections.	Diagnostic stewardship in emergency departments in South Africa	Utilization review of carbapenems, adherence to AWARE guidelines with interventions to improve.	Co-design and implementation of a nursing AMS/IPC bundle	Strengthening Primary Care Antimicrobial Stewardship Programmes across public facilities in the Western Cape: a Quality Improvement project	Dental antibiotic usage review (public & private), with interventions and monitoring of change

## Discussion

This study describes the first of four phases of a study to strengthen capacity for driving AMS implementation at scale in South Africa, based on the perspectives of health professionals actively engaged in AMS across South Africa's public and private health sectors. The goal of this first phase of the research was to provide contextual understanding for leading AMS implementation and adoption in South Africa, generate ideas for AMS interventions that the participants would co-design in Phase 2 and implement in Phase 3 and contribute to building collective will for leading AMS implementation in South Africa at scale.

The participatory action research methodology, underpinned by Theory U and graphic facilitation, enabled participants to identify systemic patterns and co-generate strategic insights in a collaborative and reflective environment. The diversity of the participant group including pharmacists, ID specialists, nurses, clinical microbiologists, IPC practitioners and AMS researchers and the cross-sectoral representation strengthened the contextual insights generated.

While the findings align with previously reported barriers and enablers to AMS implementation in South Africa^8,9,10^ the added dimensions of identifying barriers and enablers at individual, organizational/facility and provincial/national levels in South Africa have not been previously reported to the best of our knowledge.

We reflected on these factors from micro-, meso- and macro-level perspectives, namely the individual, institutional or facility, and national and provincial levels.

At the individual level, the resilience, commitment and collaborative orientation of health professionals in South Africa emerged as critical enablers. This aligns with a growing body of literature recognizing that personal qualities and leadership capabilities are foundational to AMS success.^22,23,24^ Garraghan highlights the importance of collaborative relationships between AMS leaders and key stakeholders.^22^ Similarly, So draws direct parallels between the personal qualities needed for endurance (resilience, perseverance and the ability to recover from setbacks) and those required to sustain AMS leadership over time.^23^ The professional isolation and weak interprofessional relationships identified as barriers in this study echo findings from both the South African and broader LMIC literature^8,9,14,25^ . A qualitative study of AMS implementation in public hospitals in North West Province, South Africa, found a multilevel leadership and governance vacuum operating within a dysfunctional health ecosystem, with healthcare practitioners experiencing marked dissonance between government AMS ideals and on-the-ground realities.^9^ Hierarchical tensions and poor communication across professional silos have similarly been reported in qualitative studies,^3,6,8^ underscoring that interprofessional dynamics are a near-universal challenge in AMS implementation.

At the institutional/facility level, participants identified AMS, IPC and pharmacy and therapeutics committees, access to guidelines and diagnostics and quality improvement methods as enablers. This is consistent with findings from a scoping review of hospital-based AMS in LMICs, which identified the availability of antibiotic guidelines, dedicated multidisciplinary committees and prompt laboratory support as key facilitators.^8,10,26^ However, this same body of literature has emphasized that where these structures are absent, weak, or poorly utilized, they become the most consistently cited barriers—a pattern also reflected in our study.^8,16,25,26^ A systematic review of implementation determinants for AMS in sub-Saharan Africa found that underfunded health systems, limited diagnostic and laboratory infrastructure, weak governance and insufficient training were the dominant constraints, while hospital leadership support and stakeholder engagement were among the most potent enablers.^16^ Crucially, the findings of our study suggest that in South Africa, many of these enabling structures are not absent but underutilized or inconsistently applied—a distinction with important implications for implementation strategy.

At the national and provincial level, the Essential Medicines List, the South African AMR National Strategy Framework (2018–2024), provincial AMS and IPC structures, and the advocacy role of professional societies such as SAASP, were identified as significant enablers. This is consistent with evidence from other LMIC contexts, where existing global and national frameworks have been shown to provide a foundation for AMS implementation, even when resources are constrained.^10,16,25^ However, participants also highlighted inadequate and inconsistent funding, fragmentation between public and private sectors and across provinces, and weaknesses in policy implementation as national/provincial barriers. These findings echo a South African capacity assessment, which questioned whether current resources, capacities, and leadership support were sufficient to operate AMS programmes at scale, particularly in the public sector.^6^

The findings suggest that barriers and enablers operate across levels rather than independently. At national and provincial level, limited funding, inconsistent policy implementation and fragmentation may constrain the establishment and functioning of facility-level AMS structures. Weak or under-resourced facility structures may, in turn, leave individual champions professionally isolated and dependent on personal motivation and informal relationships. Conversely, supportive national policies and professional networks may strengthen facility governance, mentorship and access to data, thereby enabling individual champions to drive local implementation. These relationships are interpretive and will require prospective examination during the implementation phases of the study.

The SWOT analysis generated through the participatory reflective process adds a further dimension to these findings, translating the multilevel barrier and enabler data into a strategic framework. The opportunities identified—particularly expanding cross-sector collaboration, harnessing existing AMS-IPC champions, leveraging technology, and initiating new ways of working within existing resource constraints—suggest that the South African AMS community is not starting from scratch, but rather building on a foundation that, while imperfect, is more substantial than the dominant discourse on LMIC barriers might suggest.^6,8,9^ The threats identified, including lack of political will, economic austerity, workforce constraints, and complacency, are formidable but not unique to South Africa and have been reported across sub-Saharan African and other LMIC settings.^10,25,26^ What is also noticeable is the relatively high weighting of strengths and opportunities which may reflect the experience from previous SAASP studies^12,13,14^ and in particular the way in which these studies address barriers and weaknesses through countermeasures in the design and execution of AMS interventions.^14^ In a recent systematic review of barriers and facilitators to AMS implementation in LMICs, Wu *et al*. highlight the importance of directly assessing the challenges posed by local context and responding to these when developing interventions.^8^ In line with this, a summary of how the findings from Phase 1 will inform subsequent phases of the SAASP Leading AMS implementation study is presented in Table 3.

**Table 3.** Summary of how Phase 1 outputs will inform subsequent phases of the SAASP Leading AMS implementation study

PHASE	PHASE 2	PHASE 3	PHASE 4
**OUTPUTS OF THE PHASE**	Training for Leading AMS implementation at scale using the BTS methodology Co-leaders will complete the design of each project during the training and prepare documents required for ethics approval of their projects.	Project co-leaders for each project will complete their call to action for their projects once ethics approval is completed. Co-leaders will implement the projects designed in Phase 2 using the BTS methods and learnings from Phase 1 and 2 of the study. The Programme Manager and Faculty mentors will continue to provide mentorship support for the implementation of the projects	Project co-leaders to capture leadership learnings and develop a framework for leading AMS implementation at scale.
			
**HOW PHASE 1 WILL INFORM THE PHASES**	Expanded content of the training derived from Phase 1: Stakeholder mapping and influencing Handling setbacks Dealing with overwhelm Change leadership key mindset shifts Leveraging enablers opportunities and strengths in design of projects Countermeasures for handling barriers, weaknesses and strengths in design of projects (examples listed in Phase 3) Improved support structures derived from Phase 1 Participants organized into MDT co-leaders for each project Faculty mentors allocated to each project Recruitment of additional subject matter experts for specific projects Added project specific sessions for 1-1 mentoring of each team by SAASP Programme Manager	We will track barrier countermeasure design elements e.g. Small increments of allocated time for interventions (time) Multifacility studies with Learning and Feedback (LFS) sessions (isolation and mindset) Training incorporated in LFS (education) Standardized data collection with testing prior to implementation (limited data) Incorporate MDT engagement (interprofessional relations) Support by subject matter experts and co-leaders of each project (education and leadership support) During the implementation phase of each project we will continue to track barriers and enablers at the three levels (individual, organizational and provincial/national) through feedback provided at overall group LFSs held every 4–6 weeks during the implementation phase. This will provide a lived experience of how the barriers and enablers hinder and/or support AMS implementation at scale that can then be reviewed with those from Phase 1.	In this phase we will reflect on all three phases of the study and will consider how the findings in Phase 1 were reflected in Phase 3 while leading the AMS implementation projects in participating public and private sector facilities in South Africa.

In Phase 2, the findings provided input for additional training content to address individual barriers and to support the project co-leaders with institutional barriers identified. In addition, examples of countermeasures for identified barriers were shared and will as far as possible be incorporated into the design of each implementation project. During Phase 3 we will also continue to track barriers and enablers that arise across all projects through the group learning and feedback sessions that will be held every 4–6 weeks. Finally, in Phase 4, the learning from the real-world implementation of the AMS projects will be considered alongside those identified in Phase 1 during the reflective workshops and incorporated into the framework for leading AMS implementation at scale.

The eight priority AMS implementation projects identified by participants reflect both the breadth of unmet need and this cohort's ambition to drive change across diverse clinical settings. The inclusion of dental and nursing-focused projects is particularly noteworthy, as these professional groups have historically been under-represented in AMS implementation research.^27^

This study has a number of limitations. Analysis was conducted by the SAASP Programme Manager, with faculty support rather than fully independent multi-coder analysis which may impact on the perspectives presented. The use of convenience sampling and reliance on a volunteer cohort responding to a SAASP call-to-action mean that the findings reflect the perspectives of health professionals already engaged in and motivated by AMS, which may introduce a positive selection bias. Data were generated through group visual mapping and reflective workshops, with no external validation, triangulation with other data sources or inclusion of dissenting perspectives. Although no external validation was achieved, we believe that we included internal qualitative triangulation. This was achieved through using multiple distinct techniques, individual journey mapping, group participative dialogues, visual multi-level barrier/enabler mapping, and a collaborative SWOT analysis, which was done to cross-verify the themes generated. Data was collected across two separate workshop locations—Cape Town and Johannesburg—with participants spanning 6 different provinces providing input from a broad geographic context. The workshops included study participants and study faculty thereby giving perspectives from different levels of expertise and seniority. While we did not capture the views of health professionals antagonistic to stewardship itself, the multidisciplinary and cross-sectoral design actively surfaced constructive dissent and professional tensions (e.g. interprofessional hierarchies, public-private fragmentations, clinician autonomy).

Nearly half of the participants were pharmacists (18 of 38) which may have influenced the perceived barriers and enablers and influenced the prioritization of the projects. This was partially offset by the inclusion of the 10 faculty members of whom seven were not pharmacists. In addition, in the workshops participants were seated in multidisciplinary groups of four or five of which only one or two were pharmacists. Existing evidence demonstrates the positive impact of pharmacists’ involvement in AMS, and as highlighted by Nampoothiri *et al.*, there needs to be a concerted effort in facilitating pharmacist roles in AMS across all countries, irrespective of income setting.^28^

A further limitation is that the findings are contextually specific to South Africa, although the identified themes resonate strongly with the broader LMIC and sub-Saharan African literature and at some level with high-income countries.^6,8,10,16,25,29^ A recent systematic scoping review compared reported barriers to AMS in 23 high income countries (HIC) and 34 LMICs and found that knowledge, education, and confidence gaps were the most frequent barrier theme in both groups.^29^ Staffing and resource constraints and organizational/governance barriers were also highly prevalent, particularly in LMIC hospitals. Workflow and documentation problems and data-system limitations were more prominent in HIC studies, whereas structural health system constraints were more prominent in LMICs.^29^ Anugulruengkitt *et al* state that implementing antibiotic stewardship can be challenging because it involves changing knowledge, deeply held attitudes and cultural norms and is influenced by the stakeholders involved.^30^ As discussed by Huttner and colleagues, sharing obstacles and success stories in implementing AMS programmes enables the global stewardship community to benefit from the experience of others.^31^

This study provides insight and context of the overall landscape for health professionals undertaking the role of leading AMS implementation at scale and opens the possibility to harness enablers, strengths and opportunities and consider countermeasures for barriers, weakness and threats in the design of the implementation projects. While potential solutions in one setting may not be transferable or even complete, we believe sharing the findings and processes may have benefits for others seeking to build capacity for AMS implementation in other geographies.

### Conclusion

This study used group participative techniques to understand the contextual landscape for AMS, to identify priorities for leading AMS implementation in South Africa and to build collective will for implementing AMS at scale. The central challenge is not to build from nothing but to identify, connect and strengthen already existing assets and to understand what countermeasures can be incorporated to address barriers, weaknesses and threats. The subsequent phases of this study, where participants will co-lead AMS projects across multiple facilities using the Breakthrough Series methodology, represent a direct response to this challenge and an opportunity to generate and test practical solutions within the realities of South Africa's health system.

## Contributors

All authors made substantial contributions to the following: (1) the conception and design of the study, or acquisition of data, or analysis and interpretation of data; (2) drafting the article or revising it critically for important intellectual content; and (3) final approval of the version to be submitted. DvdB served as Programme Manager. The members listed in the SAASP Leading AMS Implementation Study Group participated in one of the two study workshops and provided input.

## Supplementary Material

dlag232_Supplementary_Data

## Data Availability

Data are available upon request and subject to privacy, ethics and other restrictions.

## Members of the SAASP leading AMS implementation study group

Suwayda Ahmed, Anisha Chetty, Elloise du Toit, Pieter Ekermans, Elouize Fillies, Leonore Greybe, Presha Gurahoo, Chantal Hadfield, Sandi Holgate, Nicole Keuler, Sonya Kolman, Renier J Liebenberg, Tebogo Louw, Dhanya Mathew-Thomas, Zakera Mitha, Barney Mitton, Hunadi Motubatse, Samkele Mkumbuzi, Sinentokozo Mvubu, Cahlia Naested, Tamlyn Naude, Junaid Omar, Sherzana Osman, Arifa Parker, Nectarios Papavarnavas, Heidi van Reenen, Wade Rosant, Aneesah Samsodien, Nicolene Schoeman, Mariana Scheepers, Kristien Schutte, Chun Yat Chu, Veronica Ueckermann, Andriette van Jaarsveld, Lené van As, Eda Verster, Tasneem Waja, Penelope Yende.

## Supplementary data

Table S1 is available as Supplementary data at *JAC-AMR* Online.
