# Supplementary material for: Understanding the landscape for driving antimicrobial stewardship implementation at scale in South Africa
Source: JAC Antimicrob Resist. 2026 Oct 22;8(5):dlag232. doi: 10.1093/jacamr/dlag232 (PMC13687901; doi:10.1093/jacamr/dlag232)
Supplement: Supplementary file 1 [file dlag232_supplementary_data.docx]

**Supplemental Table 1**

**Consolidated criteria for reporting qualitative studies (COREQ): 32-item checklist.**

**Glass half full or half empty? Perspectives on the landscape to lead antimicrobial stewardship implementation at scale in South Africa.**

| **No. Item** | **Guide questions** | | **Description** |
| --- | --- | --- | --- |
| **Domain 1: Research team and reﬂexivity** | | | |
| ***Personal Characteristics*** | | | |
| 1. Interviewer/facilitator | Which author/s conducted the interview or focus group? | | The two workshops were facilitated by Dr Dena van den Bergh who is one of the co-investigators and the study project manager. Dr Candice Bonaconsa provided input for the design of the workshop. Dr Esmita Charani provided mentorship for the qualitative methods. |
| 2. Credentials | What were the researcher’s credentials? E.g. PhD, MD | | Dr D van den Bergh - EngD, MSc (Med), BPharm  Dr E Charani – PhD, MSc, MPharm  Dr C Bonaconsa PHD, MNursing |
| 3. Occupation | What was their occupation at the time of the study? | | Honorary Research Associate at the University of Cape Town, Department of Medicine, Division of Infectious Diseases and HIV. Independent health systems change leader and consultant. |
| 4. Gender | Was the researcher male or female? | | Female |
| 5. Experience and training | What experience or training did the researcher have? | | The interviewer has trained as an advance facilitator in Theory U and generative change leadership. She also used qualitative research during her doctoral studies and completed Presencing Foundation facilitation and training in Theory U. In addition, Drs Esmita Charani and Candice Bonaconsa who are also co-researchers for the study have extensive qualitative research experience in qualitative research in antimicrobial stewardship, provided ongoing support and mentorship for the qualitative components of the study. |
| ***Relationship with participants*** | | | |
| 6. Relationship established | Was a relationship established prior to study commencement? | | The faciitator has worked with some participants in previous collaborative studies. |
| 7. Participant knowledge of the facilitator | What did the participants know about the researcher? e.g. personal goals, reasons for doing the research | | Respondent was fully aware of the aims and objectives of the study, how the workshop was designed and would be conducted, and how the data obtained during the workshops would subsequently be used. |
| 8. Interviewer/Facilitator characteristics | What characteristics were reported about the facilitator? e.g. Bias, assumptions, reasons and interests in the research topic | | Project Manager for the research  Personal interest in AMS implementation. |
| **Domain 2: study design** | | | |
| ***Theoretical framework*** | | | |
| 9. Methodological orientation and Theory | What methodological orientation was stated to underpin the study? e.g. grounded theory, discourse analysis, ethnography, phenomenology, content analysis | Thematic content analysis of qualitative data.  Theory U framework and 3D mapping tool | |
| ***Participant selection*** | | | |
| 10. Sampling | How were participants selected? e.g. purposive, convenience, consecutive, snowball | The study participants were recruited by an open “call to participate” disseminated on LinkedIn and Instagram, through the SAASP national network and by the study faculty to their professional network. | |
| 11. Method of approach | How were participants approached? e.g. face-to-face, telephone, mail, email | Study participants were approached by faculty via email and/or telephonically. All participants were invited to attend one of four online information sessions to learn more about the study and were provided a copy of the protocol and informed consent documents. A question-and-answer session was included in the online information sessions. | |
| 12. Sample size | How many participants were in the study? | A total of 38 MDT members participated (19 pharmacists, 7 nurses and IPC practitioners, 9 doctors and/or infectious disease specialists, 4 clinical microbiologists and 1 dentist). Ten faculty members (AMR researchers and leaders) also attended one of the two workshops. | |
| 13. Non-participation | How many people refused to participate or dropped out? Reasons? | None. Participation was voluntary. | |
| ***Setting*** | | | |
| 14. Setting of data collection | Where was the data collected? e.g. home, clinic, workplace | Data was collected on visual flipchart templates at each workshop. | |
| 15. Presence of non-participants | Was anyone else present besides the participants and researchers? | No | |
| 16. Description of sample | What are the important characteristics of the sample? e.g. demographic data, date | Participants were all involved in AMS implementation in South Africa. Twenty were from the private sector and 18 from the public sector. The 38 health professionals included 19 pharmacists, 7 nurses and IPC practitioners, 9 doctors and/or infectious disease specialists, 4 clinical microbiologists and 1 dentist). Of the 10 faculty members (AMR researchers and leaders) one is from the private sector and nine are academic researchers. | |
| ***Data collection*** | | | |
| 17. Interview guide/Workshop framework | Were questions, prompts, guides provided by the authors?  Was it pilot tested? | The workshop framework was designed by the facilitator with input from faculty members.  The facilitator had previously facilitated several workshops using Theory U and 3D mapping and was supported by a faculty member (Dr Candice Bonaconsa) with previous experience in visual mapping.  No pilot was done prior to the two workshops. | |
| 18. Repeat interviews | Were repeat interviews carried out? If yes, how many? | N/A | |
| 19. Audio/visual recording | Did the research use audio or visual recording to collect the data? | No – data was collected on Visual Templates at both workshops | |
| 20. Field notes | Were ﬁeld notes made during and/or after the interview or focus group? | No additional field notes were made. | |
| 21. Duration | What was the duration of the workshops? | The two workshops were each from 09:00 to 16:00 | |
| 22. Data saturation | Was data saturation discussed? | No | |
| 23. Transcripts returned | Were transcripts returned to participants for comment and/or correction? | N/A | |
| **Domain 3: analysis and ﬁndings** | | | |
| ***Data analysis*** | | | |
| 24. Number of data coders | How many data coders coded the data? | N/A | |
| 25. Description of the coding tree | Did authors provide a description of the coding tree? | N/A | |
| 26. Derivation of themes | Were themes identiﬁed in advance or derived from the data? | The visual templates provided a framework and themes from each section was derived from the data. | |
| 27. Software | What software, if applicable, was used to manage the data? | None. Data was collated using Excel spreadsheets. | |
| 28. Participant checking | Did participants provide feedback on the ﬁndings? | No (but they shared the data on the visual templates with the larger group) | |
| ***Reporting*** | | | |
| 29. Quotations presented | Were participant quotations presented to illustrate the themes/ﬁndings? Was each quotation identiﬁed? e.g. participant number | Participant responses on the Visual template were collated and summarized.  All data was collected at a group level of 4-5 participants with no individual identification. | |
| 30. Data and ﬁndings consistent | Was there consistency between the data presented and the ﬁndings? | Yes. | |
| 31. Clarity of major themes | Were major themes clearly presented in the ﬁndings? | Yes | |
| 32. Clarity of minor themes | Is there a description of diverse cases or discussion of minor themes? | The synopsis of findings broadly touches upon the minor themes that emerged. | |
